# Influenza Vaccination Behaviour of Healthcare Workers in Switzerland: A Cross-Sectional Study

**DOI:** 10.3389/ijph.2023.1605175

**Published:** 2023-03-10

**Authors:** Phung Lang, Charlotte Tsu-Shin Wu, Anna Florence Le-Nguyen, Astrid Czock

**Affiliations:** ^1^ Department of Epidemiology, Institute of Epidemiology, Biostatistics and Prevention, Faculty of Medicine, University of Zurich, Zurich, Switzerland; ^2^ Department of Psychology, University of California at Davis, Davis, CA, United States; ^3^ QualiCCare, Baden, Switzerland

**Keywords:** prevention, healthcare workers, vaccination status, vaccination attitude, influenza vaccinations, vaccination behaviour

## Abstract

**Objectives:** As no data are available regarding the influenza vaccination status of Swiss healthcare workers (HCW) in the ambulatory setting, this study aims to investigate their influenza vaccination behaviours.

**Methods:** We conducted an online survey using a four-item, semi-structured questionnaire to assess HCWs influenza vaccination coverage and behaviour. Associations between influenza vaccination status, age and language as well as recommendation behaviour and reasons for vaccination were assessed using descriptive statistics and logistic regression analyses.

**Results:** Of the 1057 completed questionnaires, 425 (40.2%) HCW were vaccinated and 632 (59.8%) not. 78.1% of the physicians and 47.3% pharmacists were vaccinated, compared to only 29.1% of the nurses, 24.3% pharmacy technicians and 13.0% medical practice assistants (MPA). There was a significant association between influenza vaccination status and HCW profession, age, language and how often an influenza vaccination recommendation was made.

**Conclusion:** Demographic factors seem to influence HCWs’ attitudes towards influenza vaccination, which in turn affects the prospect of them recommending the influenza vaccination. Diverse strategies might be necessary to encourage HCW to get vaccinated and hence, promote influenza vaccination.

## Introduction

Vaccinations are one of the most effective public health measures to prevent the spread of infectious diseases, which is considered a human right (1).

Influenza in humans is caused by negative single-stranded RNA viruses, the influenza virus A and B of the *Orthomyxoviridae* family (2). This acute respiratory disease has been evaluated as one of the top five leading causes of illness and fatalities worldwide. Each year, it accounts for an estimated one billion infections, 3–5 million severe cases, and 290,000–650,000 influenza-related respiratory deaths globally (3, 4). Even though antivirals against influenza A and B are available, preventive immunizations are the method of choice (2). Due to antigenic variation affecting the haemagglutinin (H) and neuraminidase (N) of influenza A surface antigens, the human T- and B-cell memory cannot build up and ensure a long-lasting immunity against the disease. These variations are responsible for the annual antigenic differences, requiring a new vaccine every year (5). The fact that the inactivated vaccine, containing H and N proteins, has to be reformulated annually to match to the current circulating strains in order to be effective (2), causes annual controversial discussions with much vaccination hesitancy in the population (6).

Influenza vaccinations are recommended for different at-risk populations, especially people with chronic diseases and individuals over 65. Healthcare workers (HCW) are also recommended to get vaccinated in order to protect the vulnerable populations in their care (7). A survey of the European Centre of Disease Prevention and Control (ECDC) over eight influenza seasons states that a growing number of Member Countries recommend vaccinations for HCW; seventeen states provided coverage rates for HCW ranging from 5% to 54.9% for 2014-15 (median vaccination coverage rate was 25.7%), with varying coverage rates between years (7). The US-National Health Interview Survey (NHIS), a continuous, cross-sectional national household survey, revealed for 2015 an increase of influenza vaccination coverage among non-institutionalized US citizens aged 19 and above by 1.6% points to 44.8% compared to the previous season (8).

Studies on the benefit of HCW being vaccinated as well as their vaccination coverage against influenza are diverse, small or of low quality and show mixed evidence. Nevertheless, the importance of HCW being vaccinated against influenza is indisputable (9–14). Worldwide, the vaccine uptake among HCW varies widely—from less than 5%–80%, depending on the country (15). In Europe, overall influenza vaccination rates remain below 40% with only the Netherlands achieving the recommended 75%, and vaccination remains voluntary amongst HCW (16, 17). Attitudes toward influenza vaccination in Switzerland have historically been low, with influenza vaccination rates level around 15% (18).

Switzerland’s population was approximately 8.4 million people in 2016 (19). With 26 cantons, Switzerland has three primary linguistic regions: the German-speaking (the majority), the French-speaking, and the Italian-speaking region. To our knowledge, no data was available on the influenza vaccination behaviour of HCW working mainly in the Swiss ambulatory setting; hence, we designed a study to quantitatively evaluate their vaccination status and their likelihood to recommend influenza vaccinations to their patients.

## Methods

### Data Collection

This cross-sectional study was conducted in autumn 2016, concerning the influenza season 2015/16. Data was collected anonymized at the individual level using a semi-structured online-questionnaire in three of the four official Swiss languages - German, French and Italian.

Assuming an influenza vaccination coverage of 30% among HCW and a population size of approximately 77,800 HCW (20–25), we calculated that a sample size of 323 participants was needed.

The target population consisted of HCW in an ambulatory setting, namely physicians, pharmacists, nurses, medical practice assistants (MPA) and pharmacy technicians as well as other HCW in diverse functions. Participants were recruited *via* participating national professional societies, healthcare leagues and medical networks (HMOs) who sent a short descriptive text about the survey and the links to the online questionnaires in a single newsletter or direct electronic mailing to their members. To ensure absolute anonymity of the data, the online platform Survey Monkey was set up for the responses to not be traceable to the source of input. Participation was voluntary and the mailing was done only once. By responding to the survey, the participants consented to the fully anonymized use of the submitted data for analysis. The questionnaire was available online during the month of November 2016.

An enquiry to the Ethics Committee confirmed that the study did not fall within the scope of the Human Research Act (HRA) and authorization was not required (BASEC Nr. Req-2023-00101).

### Questionnaire

The questionnaire contained a demographic section and four items regarding the study objective. The demographics age, sex and healthcare profession/function were collected. The four items included: 1) influenza vaccination status during the preceding winter 2015-16, 2) reasons for getting/not getting an influenza vaccination, 3) how often they recommended the influenza vaccination to their patients and 4) reasons for and not recommending the influenza vaccination (Supplementary S1- Questionnaire).

### Analysis

To calculate the response rate, we used the membership information published on the website of the participating health professional organizations (20–25). The raw data from the survey was quantified into the following categories: profession, sex, language, age, vaccination status and the reasoning as well as how often vaccination was recommended to their patients and the respective reasoning for their action. Only cases with completed data for language, age, sex and vaccination status were retained for analysis. To calculate the proportion of participants 60 years and older, we defined a cut-off age of 70 since the professional organizations sent the mailing with the survey link to their active members and employees. Although the regular retirement age in Switzerland was 65 for men and 64 for women in 2016, this seldom applies to the HCW in the ambulatory setting, in particular to physicians and pharmacists. Language was defined according to the language of the completed questionnaire. Pearson Chi-squared tests of independence were performed to evaluate the associations between influenza vaccination status and HCW profession, age, language, and the prospect of recommending influenza vaccination. A multivariable logistic regression analysis was conducted to evaluate the association between the odds of being vaccinated against influenza and sex, age, language and profession; crude and adjusted odds ratios (OR) and 95% confidence intervals were calculated. *p*-value <0.05 was considered significant. All data analysis was performed using STATA Version 14.2.

## Results

### Participant Characteristics

In total, 1063 (1.4%) HCW completed the questionnaire. 3 cases did not indicate their influenza vaccination status and sex was missing in 3 cases, leaving 1057 cases for further analysis. Among these were 11 different HCW professions, with highest participation seen in pharmacists (41.0%), physicians (14.3%), pharmacy technicians (13.2%), nurses (10.4%) and MPA (9.5%) (Table 1). The geographical and age distributions of the participants corresponded to the different linguistic regions and age categories presented in Switzerland.

**TABLE 1:** Distribution of the study participants aged 16+ detailed by profession, linguistic region and age and proportion vaccinated against influenza, Switzerland, 2016.

Demographic variable	N	%	N (%) Vaccinated	Swiss population 2016 (%)^a^
Profession				
Physicians	151	14.3	118 (78.1)	-
Pharmacists	433	41.0	205 (47.3)	-
Nurses	110	10.4	32 (29.1)	-
Psychosocial Counsellors	16	1.5	4 (25.0)	-
Pharmacy Technicians	140	13.2	34 (24.3)	-
Other Health Professions	24	2.3	5 (20.8)	-
Management/. Administration	40	3.8	8 (20.0)	-
Other	28	2.6	5 (17.9)	-
MPAs	100	9.5	13 (13.0)	-
Prevention/Public Health Officers	13	1.2	1 (7.7)	-
Other Medical Professions	2	0.2	0	-
Total	1057	100	425 (40.2)	-
Sex				
Female	793	75.0	275 (34.7)	49.6
Male	264	25.0	150 (56.9)	50.4
Total	1057	100	425 (40.2)	
Language of HCW				
French-speaking	289	27.3	139 (48.1)	25.8
German-speaking	713	67.5	266 (37.3)	70.1
Italian-speaking	55	5.2	20 (36.4)	4.1
Total	1057	100	425 (40.2)	
Age group				
16–20	60	5.7	4 (6.7)	7.4
21–30	203	19.2	57 (28.1)	17.9
31–40	215	20.3	89 (41.4)	19.5
41–50	226	21.4	92 (40.7)	20.6
51–60	274	25.9	134 (48.9)	20.0
61+	79	7.5	49 (62.0)	14.7
Total	1057	100	425 (40.2)	

^a^ 2016 Population data from Swiss Federal Statistical Office [47]. Chi-squared test of independence between influenza vaccination status and different HCWs: Pearson Chi-squared (10) = 175.7419, *p* = 0.000; language of the HCW: Pearson Chi-squared (10) = 10.315, *p* = 0.006; and age: Pearson Chi-squared (5) = 64.9113, *p* = 0.000.

n, number of participants; HCW, healthcare professional.

### Vaccination Status by Profession, Language and Age

There was a significant difference in the vaccination coverage across the 11 HCW professions (Pearson Chi-square = 175.74, *p* = 0.000): 78.1% of physicians, 47.3% of pharmacists, 29.1% of nurses, 25.0% of psychosocial counsellors, 24.3% of pharmacy technicians, 20.8% of other health professions, 20% of management/administration, 13% of MPA, 7.7% of prevention/public health officers and 17.9% of Others being vaccinated against influenza. Two participants identified as Other Medical Professions were not vaccinated.

In Table 1, we also see that there is a significant relationship between language and vaccination status (Chi-square = 10.32, *p* = 0.006), where 37.3% of German-speaking participants reported receiving the influenza vaccination. With the French-speaking participants, this figure was 48.1% and with Italian-speaking participants 36.4%. Supplementary S2 shows that among the 11 HCW professions, the French-speaking HCW tend to have a better influenza vaccination coverage than the German-speaking HCW.

We further examined the relationship between profession, language and vaccination status. Based on the number of participants, this analysis was only conducted for physicians, nurses, pharmacists and pharmacy technicians (Supplementary S2). There was a significant relationship between language and vaccination status among pharmacy technicians: Chi-square = 22.95, *p* = 0.000) and physicians: Chi-square = 10.04, *p* = 0.007; in contrast, there was no significant association between language and likelihood of vaccination among pharmacists (Chi-square = 2.10, *p* = 0.350) and nurses (Chi-square = 2.38, *p* = 0.123).

A multivariable logistic regression analysis shows the association between the odds of being vaccinated against influenza and age, sex, language of the HCW and profession (Table 2). Physicians and pharmacists have significantly higher odds of being vaccinated against influenza than nurses (Adjusted Odds Ratio (AOR): 7.73 (95% CI 4.28–13.96) and 2.07 (95% CI 1.29–3.31), respectively). Furthermore, French-speaking HCW have significantly higher odds of being vaccinated than their German-speaking counterparts (AOR: 1.77 (95% CI 1.29–2.42)). There is also a significant relationship (Chi-square = 64.91, *p* = 0.000) between age and vaccination status (Tables 1, 2). The older the participant, the higher the prevalence of influenza vaccination; conversely, younger participants exhibited lower vaccination rates.

**TABLE 2:** Adjusted participant characteristics associated with influenza vaccination status using logistic regression, Switzerland, 2016.

	n	Crude odds ration	95% CI lower	95% CI upper	*p*-value	Adjusted odds ratio	95% CI lower	95% CI upper	*p*-value
Language									
German	713	Ref	—	—	—	Ref	—	—	—
French	289	1.56	1.18	2.05	0.002	1.77	1.29	2.42	0.000
Italian	55	0.96	0.54	1.70	0.889	0.75	0.41	1.38	0.356
Sex									
Female	793	Ref				Ref			
Male	264	2.48	1.87	3.29	0.000	1.29	0.92	1.81	0.141
Age group									
16–20	60	Ref	—	—	—	Ref	—	—	—
21–30	203	5.47	1.89	15.77	0.002	3.44	1.16	10.25	0.026
31–40	215	9.89	3.46	28.26	0.000	4.31	1.43	12.98	0.009
41–50	226	9.61	3.37	27.43	0.000	4.01	1.32	12.19	0.014
51–60	274	13.40	4.73	37.97	0.000	5.11	1.69	15.45	0.004
60+	79	22.87	7.52	69.49	0.000	5.94	1.80	19.58	0.003
Profession									
Nurse	110	Ref	—	—	—	Ref	—	—	—
MPA	100	0.36	0.18	0.74	0.006	0.61	0.29	1.30	0.202
Physician	151	8.72	4.96	15.32	0.000	7.73	4.28	13.96	0.000
Pharmacist	433	2.19	1.39	3.45	0.001	2.07	1.29	3.31	0.002
Pharmacy Technician	140	0.78	0.44	1.37	0.393	0.99	0.54	1.80	0.971
Others	123	0.56	0.30	1.03	0.064	0.54	0.29	1.01	0.053

n, number of participants; Total n, 1057; Ref, reference; CI , confidence interval.

### Reasons for Vaccination Among HCWs

**FIGURE 1:**
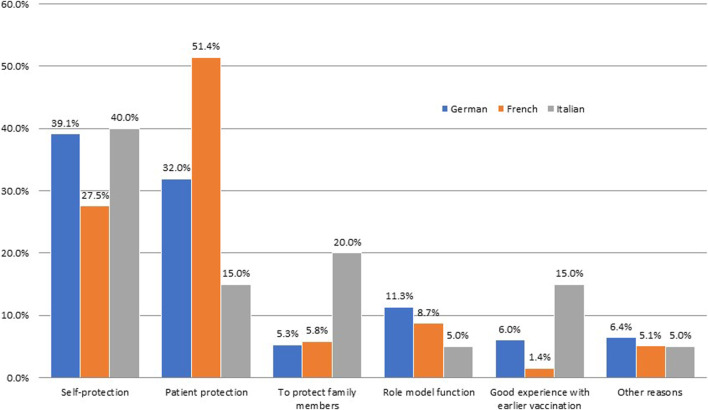
Reasons for getting influenza vaccination detailed by language of the healthcare worker, Switzerland, 2016. HCW = healthcare worker. n = German-speaking: 266; French-speaking: 138; Italian-speaking: 20. Pearson Chi-squared test for independence (10) = 31.7705, *p* = 0.000 between language of HCW and reasons for HCW getting vaccinated against the influenza.

In the questionnaire, five reasons to be vaccinated against the influenza were listed: self-protection, patient protection, to protect family members, role model function, good experience with earlier vaccination, or another specific reason (Figure 1). Of the 138 French-speaking HCW, 51.4% were vaccinated for patient protection. Of the 266 German-speaking HCW, 39.1% were vaccinated for self-protection and 32.0% for patient protection. Of the 20 Italian-speaking HCW, 40.0% were vaccinated for self-protection and 20.0% for protecting family members. There was a significant relationship between language and reason for vaccination among HCW (Chi-square = 31.77, *p* = 0.000).

Fourteen reasons not to be vaccinated against the influenza were also listed on the questionnaire (Supplementary S3). Of these reasons, fear of side effects (11.0%) and self-determination of getting the influenza vaccination (11.1%) were cited the most. 185 “other” reasons were also stated by the participants, of which the most common was they perceived themselves being healthy (40.0%) and the use of homeopathy/alternative medicine (10.8%). The different HCW who elected not to get vaccinated had individualized reasons and no pattern was detectable. Hence, no significant associations could be detected between language and reasons for not vaccinating.

### Recommendation of an Influenza Vaccination to Their Patients

Table 3 shows a significant relationship between the type of HCW and the prospect of them recommending vaccination to their patients (Chi-square = 190.48, *p* = 0.000). Physicians, pharmacists, nurses and MPAs most likely either always recommended vaccination to their patients or only in certain cases (99.3%, 98.3%, 97.2% and 96.9%, respectively). Of these four HCW, 67.3% of physicians, 61.8% of pharmacists and 57.4% of nurses always recommended vaccination to their patients compared to only 43.3% of MPA.

**TABLE 3:** Healthcare worker category and whether they always, never, or only recommend the influenza vaccine to their patients in certain cases, Switzerland, 2016.

Profession	n (%)	Always recommend (%)	Recommend in certain cases (%)	Never recommend (%)
Physicians	150 (14.4%)	101 (67.3%)	48 (32.0%)	1 (0.7%)
Pharmacists	427 (41.1%)	264 (61.8%)	156 (36.5%)	7 (1.6%)
Nurses	108 (10.4%)	62 (57.4%)	43 (39.8%)	3 (2.8%)
Psychosocial Counsellors	16 (1.5%)	8 (50.0%)	6 (37.5%)	2 (12.5%)
Pharmacy Technicians	138 (13.3%)	38 (27.5%)	85 (61.6%)	15 (10.9%)
Other Health Professions	23 (2.2%)	7 (30.4%)	12 (52.2%)	4 (17.4%)
Management/Administration	39 (3.8%)	12 (30.8%)	16 (41.0%)	11 (28.2%)
Other	27 (2.6%)	11 (40.7%)	8 (29.6%)	8 (29.6%)
MPAs	97 (9.3%)	42 (43.3%)	52 (53.6%)	3 (3.1%)
Prevention/Public Health Officers	13 (1.3%)	2 (15.4%)	6 (46.2%)	5 (38.5%)
Other Medical Professions	2 (0.2%)	0 (0%)	2 (100%)	0 (0%)
Total	1040 (100%)	549 (52.6%)	435 (41.7%)	59 (5.7%)

n = number of participants = 1040. Chi-squared test of independence between type of HCW and prospect of influenza vaccination recommendation to their patients: Pearson Chi-squared (20) = 190.4773, *p* = 0.000.

**FIGURE 2:**
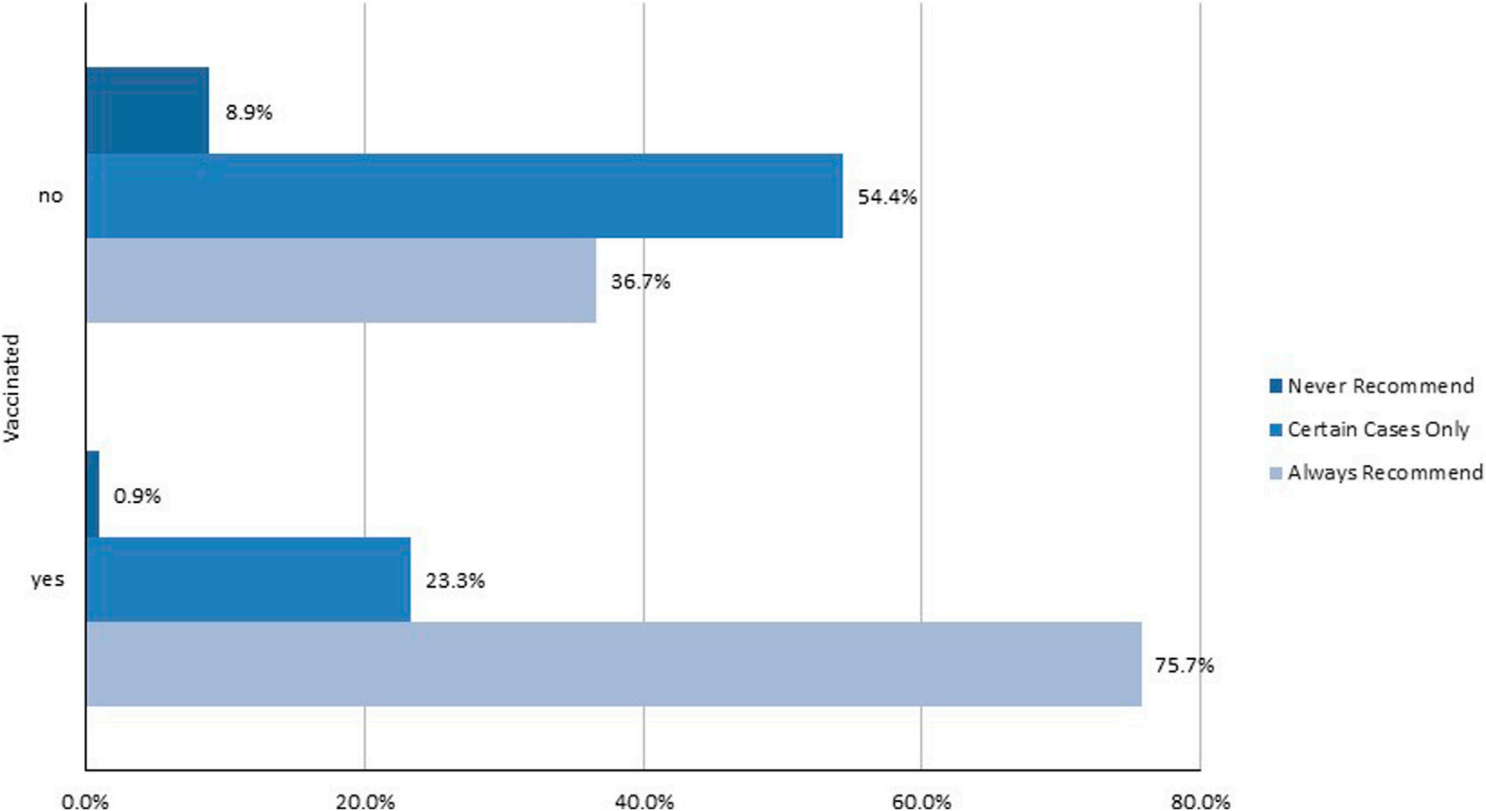
Influenza vaccine recommendation to patients based upon healthcare worker vaccination status, Switzerland, 2016. HCW = healthcare worker. n = not vaccinated: 616; vaccinated: 424. Pearson Chi-squared test for independence (2) = 158.8847, *p* = 0.000 between HCW vaccination status and influenza vaccination recommendation to their patients.

A closer examination between the vaccination status of the HCW and the prospect they recommended an influenza vaccination to their patients showed a significant association (Chi-square = 158.88, *p* = 0.000). Of the 616 HCWs who were not vaccinated, 8.9% never recommended vaccination to their patients, 54.4% did so only in certain cases and 36.7% always recommended vaccination. When looking at the 424 HCWs who were vaccinated, 0.9% never recommended vaccination to their patients, 23.3% did so only in certain cases and 75.7% always recommended vaccination (Figure 2).

**FIGURE 3:**
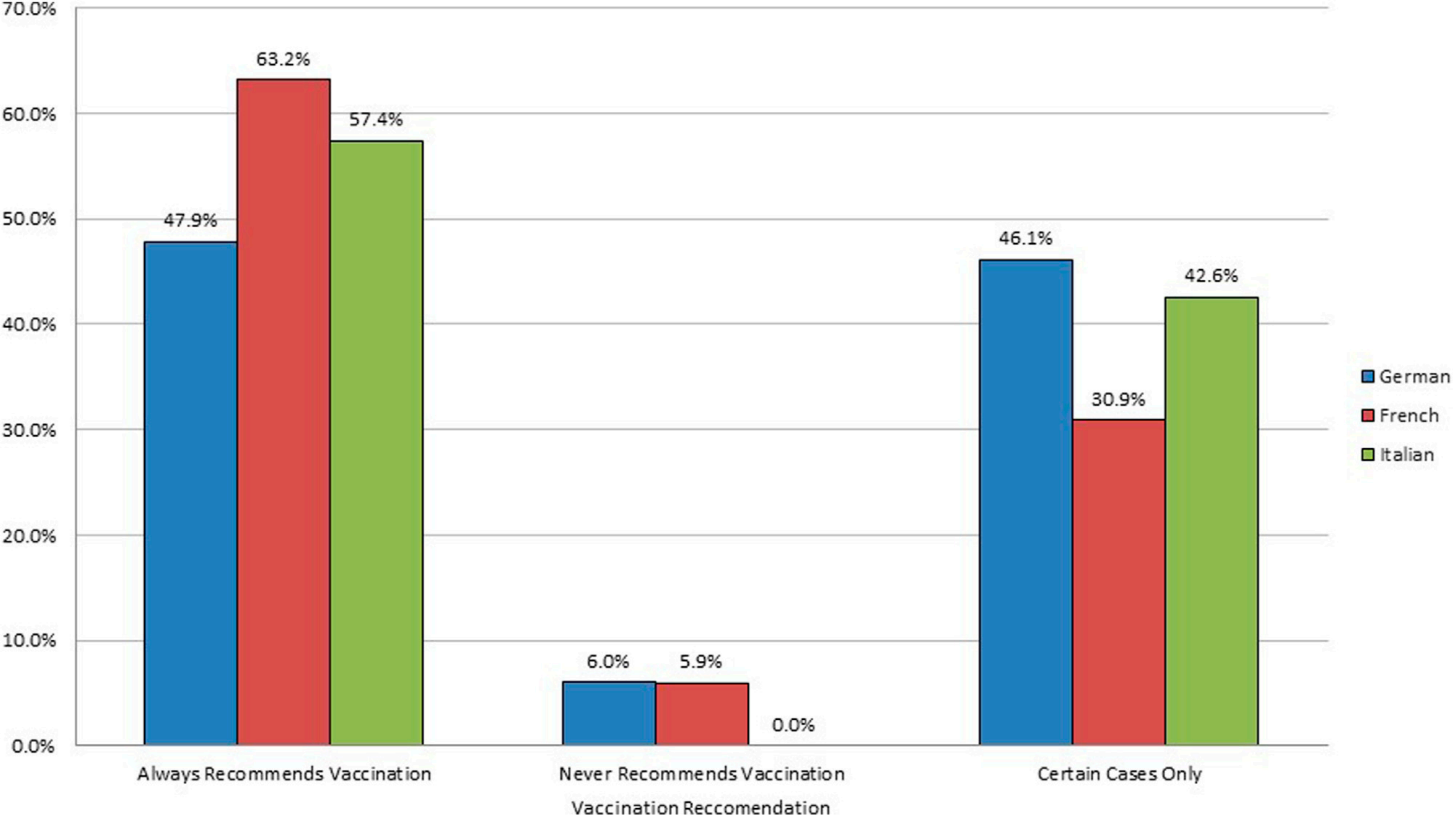
Language of the healthcare worker versus influenza vaccination recommendation to patients, Switzerland, 2016. HCW = healthcare worker. n = German-speaking: 698; French-speaking: 288; Italian-speaking: 54. Pearson Chi-squared test for independence (4) = 23.9531, *p* = 0.000 between language of HCW and influenza vaccination recommendation to their patients. Influenza vaccination behaviour of healthcare workers in Switzerland: a cross-sectional study.

We also observed a significant relationship between language and the prospect of recommending vaccination to their patients (Chi-square = 23.95, *p* = 0.000). Among 698 German-speaking HCWs 47.9% always recommended vaccination to patients, 6.0% never recommended vaccination, and 46.1% recommended vaccination only in certain cases. Out of 288 French-speaking HCWs, 63.2% always recommended vaccination to patients, 5.9% never recommended vaccination, and 30.9% recommended vaccination only in certain cases. With the 54 Italian speaking HCWs, 57.4% always recommended vaccination to patients, 0% never recommended vaccination, and 42.6% recommended vaccination only in certain cases (Figure 3).

## Discussion

This study offers new insights into the vaccination acceptance of the influenza for a broad range of healthcare providers in Switzerland. Within the examined population, age, profession and language proved to be the most influential variables affecting HCWs’ behaviour toward annual influenza vaccinations for themselves and their patients across Switzerland. A 2018 study across six European countries showed that profession, country of origin, and age are three major factors affecting attitudes towards vaccination (26).

### Vaccination Status by Profession, Language and Age

Among the 40.2% of the HCW included in our study that were vaccinated, physicians (78%) were most likely to be vaccinated, followed by pharmacists (47%), nurses (29%), pharmacy technicians (24%) and MPAs (13%), who appeared to be more critical of vaccinations. Various international studies have confirmed this trend, with a closer examination in the following case studies: A study in Italy confirmed a significant global vaccination hesitancy in nurses, finding far lower than optimal vaccine rates among nurse practitioners (27), and a study in Switzerland confirmed a trend in nurses’ vaccine distrust (28).

Compared to German- and Italian-speaking HCW, French-speaking HCW in Switzerland were more likely to be vaccinated. French-speaking pharmacy technicians and physicians had a higher prospect of being vaccinated compared to their German- and Italian-speaking counterparts. This observation corresponded with the findings of a previous study that concluded that the HCWs in French speaking-regions of Switzerland had the highest influenza vaccination coverage (29). It is however worth noting that, among pharmacists and nurses, there was no significant data to prove different regional vaccination rates. A recent study showed that German-speaking Swiss dental healthcare workers had higher awareness about vaccine preventable infectious diseases than their French-speaking counterparts (30).

The average age of HCW was a notable factor in vaccination rates, with distinct vaccination behaviour discrepancies among the various age ranges. Younger HCW were less likely to get the yearly influenza due to feeling stronger and healthier, an attitude corroborated by a study carried out in Turkey after the H1N1 influenza pandemic in 2009 (31). A major reasoning behind this attitude was that young healthy adults often primarily attributed their protection against diseases to their good health; furthermore, the lack of vaccine information increased the tendency of their decision not to get immunized (32). This attitude is also present internationally, with research in the U.S. demonstrating a widespread belief (in young adult civilians and HCWs alike) that good health is enough protection, with vaccination being unnecessary (33). As HCW age increased, the prospect of annual influenza vaccination increased steadily. This is interesting, since the antibody response in older/elderly people is lower than in young healthy adults (34). This further indicates the necessity of younger people getting vaccinated.

### Reasons for Vaccination

As confirmed in previous studies (35, 36), self-protection, patient protection, and protecting family members were the most prominent reasons why HCW got vaccinated across all three regions. French-speaking HCW most often cited first patient protection and then self-protection. Italian-speaking HCW most often reasoned with self-protection first and protecting family members second. German-speaking HCW most often stated self-protection first and then patient protection. Alongside the three aforementioned prominent reasons, wanting to set a positive example for others was another reason why HCW were getting vaccinated. These reasons show the importance of having HCW getting vaccinated, in addition to their increased tendency of recommending vaccination to their patients, as shown in our results and elsewhere (35). There were no significant reasons for those who chose not to get vaccinated. The reasons varied equally, with a lack of knowledge about the vaccine and distrust over vaccine efficacy, which have also been confirmed in a recent systematic review about HCW vaccine perceptions and hesitancy (35). A lack of evidence-based information, as well as personal autonomy, proved to be the biggest barriers against deciding to get influenza vaccinations amongst the general population, and a study has shown how there were even common themes prevalent globally amongst those who doubt the vaccine (37).

### Recommendation of Influenza Vaccination to Their Patients

HCW who were vaccinated were more likely to recommend vaccination for their patients, which was supported by another study on immunization attitudes of vaccinated physicians (38). 75.8% of vaccinated HCW always recommended vaccination to their patients while only 36.7% of unvaccinated HCW did. The results of this study were confirmed in another study (39) showing vaccinated HCW recommending the vaccine, versus a lower percentage of unvaccinated HCW recommending it. While HCW did not feel the need to get vaccinated themselves, their opinions on the matter continually affected the general public, as HCW behaviours towards vaccination played a key role in patient decision-making to get the shot (35, 40, 41). Our study also showed that French-speaking HCW were also more likely to consistently recommend vaccinations to their patients as opposed to German- and Italian-speaking HCWs. This evidence could suggest cultural differences on vaccination attitudes, a fact also addressed in other studies (6, 42).

### Limitations and Areas of Further Research

One limitation of this study is the unknown response rate due to the recruiting method, as the participating organisations sent out the link to all their members but the exact number of recipients and people who read the newsletter or direct mailing could not be assessed. Therefore, we could not determine the representativeness of the results. As we also relied on the distribution of the questionnaire-links on professional societies and other organisations to recruit participants, we cannot guarantee that only HCWs from the ambulatory sectors participated in the study. However, the participating professional societies and the other participating organisations (health league, HMO, pharmacy chain) comprise of mainly HCW from the ambulatory setting or work exclusively in the ambulatory setting, respectively. Additionally, selection bias was highly possible, as participants who were more likely to have a positive attitude towards vaccinations or were more interested in the topic would respond. For example, as the participating organizations were members of the non-profit association QualiCCare and therefore known for their investment in quality of care, their members might have also shown a higher affinity to the investigated topic. Additionally, the high participation of female HCW in this study could have biased the results as female HCW have a higher tendency to provide or recommend vaccinations to their patients (18). However, in our logistic regression analysis, after adjusting for sex, language, age and profession, there was still a significant association between the odds of being vaccinated against influenza and language, age and profession. Finally, due to ease, cost and speed, the survey was performed online, but we recruited participants through targeted organizations. No questionnaires in French or Italian were answered by MPA, which could have introduced a negative selection bias as the MPA from the German-speaking region were specifically invited by their direct employers to answer with the assurance that the answers were completely anonymous and could not be retraced to their origin. A comparison of the participants to the general population showed similar demographic distribution by linguistic region and age, although the language of the questionnaire was used as a proxy for linguistic region because the place of residence was not collected. Moreover, many of the results we obtained for Switzerland corresponded to the published national and international literature on this topic (18, 26, 27, 30, 35).

Although the questionnaire was not pre-tested for reliability and validity prior to its distribution, the evaluation of the responses showed no misleading questions. Our survey could serve as a pilot study which could be used to develop further research with a larger sample size overall (and regionally within all Swiss Cantons) to gather more data on HCW attitudes towards the influenza vaccination, its effect on the general population, and their vaccination uptake.

As this study occurred prior to the COVID-19 pandemic, it would be beneficial to examine how attitudes and behaviours towards vaccinations have changed in Switzerland with the new global pandemic (43, 44). HCW remain perhaps the most influential advisors of public vaccine decisions, therefore their support is crucial in trying to increase future vaccinations (40, 45, 46). Updated studies on HCW attitudes towards vaccinations provide essential data that aids future disease-prevention courses of action in large healthcare settings, in addition to helping future vaccination promotion campaigns. This study is especially significant in the context of the COVID-19 pandemic, given how the most effective solution to overcoming the pandemic is a worldwide vaccination effort. Therefore, it is imperative to develop a greater understanding of HCWs’ attitudes towards vaccinations. This information will prove invaluable for anticipating the actions of HCW during future global pandemics, allowing for planning more effective pandemic responses.

### Conclusion

Our study analysed different factors that could affect the vaccination behaviours of HCW, which can be ultimately used to positively affect the populations’ attitudes toward influenza vaccination. Demographic factors seem to influence the HCWs’ attitudes towards influenza vaccination, which in turn affects the prospect of them recommending the influenza vaccination. Diverse strategies might be necessary to encourage HCW to get vaccinated and hence, promote influenza vaccination.
