# Supplementary material for: Influenza Vaccination Behaviour of Healthcare Workers in Switzerland: A Cross-Sectional Study
Source: Int J Public Health. 2023 Mar 10;68:1605175. doi: 10.3389/ijph.2023.1605175 (PMC10036349; doi:10.3389/ijph.2023.1605175)
Supplement: Supplementary file 1 [file DataSheet1.pdf]

Influenza vaccination behaviour of healthcare workers in Switzerland: a cross-sectional study

Supplementary 1:

Standardized questionnaire for professionals working in primary care practices / pharmacies / health care organizations / association / leagues / health insurance companies, Switzerland, 2016

Influenza vaccination: what do you think about it?

The annual influenza vaccination, as you probably know, is a controversial topic every year. We are interested in hearing your opinion about it. Your responses will be treated anonymously. By participating in the survey, you will help us better analyse the influenza vaccination preparedness. Thank you.

Demographics

Age:
☐ 16 – 20
☐ 21 – 30
☐ 31 – 40
☐ 41 – 50
☐ 51 – 60
☐ 61 or older

Gender: ☐ male ☐ female

Function/profession:

☐ physician
☐ pharmacist
☐ other medical professional according to the Swiss Law of Medical Professions (dentist, chiropractor, veterinarian)
☐ registered nurse
☐ other health professional / therapist (Physiotherapist, nutritionist, diabetes nurse, ..)
☐ medical practice assistant
☐ pharmacy technician
☐ psychosocial therapist
☐ Administration
☐ Prevention / Public Health
☐ other ……….

Questions

1. Did you get vaccinated against influenza last winter?

☐ yes ☐ no

If yes: why?

☐ patient protection
☐ personal protection
☐ good experience with prior vaccinations
☐ to protect family members
☐ role model
☐ other reasons, namely: …………………………………………………………………

multiple answers possible

If no: why not?

☐ fear of common side effects
☐ increased susceptibility to infections
☐ influenza infection due to vaccination
☐ influenza infection despite of vaccination
☐ hygiene measures and masks are sufficient
☐ vaccination does not work / is too weak
☐ Won't let anyone tell me what to do / self-determination
☐ the visitors / family members are the problem (of infection)
☐ I stay at home when I am sick
☐ an influenza infection boosts the immune system
☐ influenza is harmless
☐ patients should vaccinate themselves
☐ the vaccination is not sufficiently researched
☐ Distrust of pharmaceutical industry
☐ other reasons, namely:…………………………………………………………………

Multiple answers possible

2. **Do you recommend you patients / clients the vaccination against the seasonal influenza?**

☐ yes ☐ no ☐ only in certain cases

If yes: Why?

☐ by conviction
☐ to protect my patients / clients
☐ other reasons, namely:…………………………………………………………………

If not: Why not?

☐ I am not convinced of the influenza vaccination
☐ I do not get vaccinated myself
☐ this is a private matter / autonomy
☐ This is the matter of the treating physician

☐ other reasons, namely:………………………………………………………………

If only in certain cases: In which cases?

☐ with patients at risk

☐ when I get asked

☐ other reasons, namely:………………………………………………………………

3. Do you know the electronic vaccination card, www.meineimpfungen.ch?

☐ yes

☐ no

4. If you know it: do you use the electronic vaccination card www.meineimpfungen.ch?

☐ yes

☐ No: why not?

- ☐ no time / too much effort
- ☐ too little use
- ☐ too complicated
- ☐ no internet access
- ☐ doubt data protection
- ☐ other reasons, namely:………………………………………………………………

03.10.2016 /

Supplementary 2.

Distribution of profession by linguistic region and proportion vaccinated for the influenza, Switzerland, 2016.

Profession	n	n by Language	% Vaccinated by Language
Pharmacists	433	253 German<br>132 French<br>48 Italian	48.6% German<br>48.5% French<br>37.5% Italian
Doctors/ Physicians	151	122 German<br>28 French<br>1 Italian	73% German<br>100% French<br>100% Italian
Pharmacy Technicians	140	82 German<br>55 French<br>3 Italian	9.8% German<br>45.5% French<br>33.3% Italian
Nurses	110	80 German<br>30 French<br>0 Italian	25.0% German<br>40.0% French<br>-
MPAs	100	100 German<br>0 French<br>0 Italian	13% German<br>-<br>-
Management/Administration	40	25 German<br>15 French<br>0 Italian	12% German<br>33.3% French<br>-
Other	28	15 German<br>11 French<br>2 Italian	33.3% German<br>0% French<br>0% Italian
Other Health Professions	24	11 German<br>12 French<br>1 Italian	9.1% German<br>33.3% French<br>0% Italian
Psychosocial Counselors	16	14 German<br>2 French<br>0 Italian	21.4%German<br>50% French<br>-
Prevention/ Public Health Officers	13	9 German<br>4 French<br>0 Italian	13%German<br>0% French<br>n/a
Other Medical Professions	2	2 German<br>0 French<br>0 Italian	0% German<br>-<br>-
Total	1057	713 German<br>289 French<br>55 Italian	37.3% German<br>48.1% French<br>36.4% Italian

Supplementary 3:

Table of Reasons for not getting the influenza vaccination (%), Switzerland, 2016

Reason for not vaccination	German	French	Italian	Total
Other reasons	32.4	29.9	26.5	30.3
Fear of common side effects	11.0	10.6	14.7	11.0
Won't let anyone tell me what to do / self-determination	10.3	10.0	29.4	11.1
I stay at home when I am sick	7.6	7.6	5.9	7.5
Vaccination does not work / is too weak	7.6	7.2	0.0	6.9
Influenza despite the vaccination	2.1	8.3	5.9	6.7
Increased susceptibility to infections	1.4	8.3	2.9	6.4
Distrust of pharmaceutical industry	10.3	4.6	2.9	5.9
an influenza infection boosts the immune system	3.4	3.5	0.0	3.3
Influenza is harmless	3.4	3.5	2.9	3.4
Hygiene measures and mask are sufficient	5.5	3.9	5.9	4.4
the vaccination is not sufficiently researched	0.0	1.9	2.9	1.5
Patients should vaccinate themselves	4.8	0.7	0.0	1.6
The relatives/visitors are the problem (contagion)	0.0	0.0	0.0	0.0
Influenza infection due to vaccination	0.0	0.0	0.0	0.0
Total	100.0	100.0	100.0	
